# Proposed flowchart for the management of suspected work-related mental disorders: an experience report

**DOI:** 10.47626/1679-4435-2025-1395

**Published:** 2025-12-19

**Authors:** Ana Cleide Silva Dias, Rene Elias Gonçalves

**Affiliations:** 1 Faculdade de Medicina, Universidade Federal do Vale do São Francisco (Univasf), Petrolina, PE, Brazil

**Keywords:** mental disorders, work, primary health care, workflow, mental health services., | transtornos mentais, trabalho, atenção primária à, saúde, fluxograma, serviços de saúde mental.

## Abstract

**Introduction:**

Work-related mental disorders are triggered by factors present in the work environment. Primary health care professionals often face difficulties in establishing the causal link and managing these cases appropriately, largely due to the lack of tools to guide clinical care and referral.

**Objectives:**

To report the experience of students and faculty in the participatory development of a flowchart for treating suspected Work-Related Mental Disorders cases, involving professionals from the specialized Occupational Health and Mental Health services of the Juazeiro Municipal Health Department, Bahia.

**Methods:**

This descriptive experience report was prepared in 2023 as part of an outreach project linked to the Education through Work for Health Program.

**Results:**

The development of the flowchart was organized into four stages: i) technical visits to the Psychosocial Care Center and to the Reference Center for Worker’s Health, aimed at bringing teams closer and understanding routine care processes; ii) presentation and discussion, in a dialogue circle, of the Mental Health and Work Care Protocol and the Notifiable Diseases Information System form, focusing on the identification of gaps; iii) collective analysis of the existing care pathway, which revealed weaknesses in communication between services; and iv) shared construction of the flowchart, using active-learning strategies to stimulate critical thinking and strengthen the leadership of the professionals involved.

**Conclusions:**

The multidirectional and participatory flowchart increased professionals’ awareness, improved intersectoral communication, and strengthened coordination between Worker’s Health and Mental Health services, facilitating the identification and referral of suspected cases.

## Abstract (pt)

**Introdução:**

Os transtornos mentais relacionados ao trabalho são desencadeados por fatores presentes no ambiente laboral. Profissionais da atenção primária à saúde frequentemente enfrentam dificuldades para estabelecer o nexo causal e conduzir adequadamente esses casos, em razão da escassez de instrumentos que orientem o atendimento e o encaminhamento.

**Objetivos:**

Relatar a experiência de alunos e docentes na construção participativa de um fluxograma para atendimento de casos suspeitos de transtornos mentais relacionados ao trabalho, desenvolvido em colaboração com profissionais dos serviços especializados em Saúde do Trabalhador e Saúde Mental da Secretaria Municipal de Saúde de Juazeiro, estado da Bahia, Brasil.

**Métodos:**

Relato de experiência, de caráter descritivo, elaborado em 2023 no âmbito de um projeto de extensão vinculado ao Programa de Educação pelo Trabalho para a Saúde.

**Resultados:**

O processo de construção do fluxograma foi organizado em quatro etapas: i) realização de visitas técnicas ao Centro de Atenção Psicossocial e ao Centro de Referência em Saúde do Trabalhador, com o objetivo de aproximar as equipes e reconhecer as rotinas assistenciais; ii) apresentação e discussão, em roda de conversa, do Protocolo de Atenção à Saúde Mental e Trabalho e da ficha do Sistema de Informação de Agravos de Notificação, com foco na identificação de lacunas; iii) análise coletiva do fluxo assistencial existente, evidenciando fragilidades na comunicação entre os serviços; e iv) elaboração compartilhada do fluxograma, utilizando metodologias ativas para estimular o pensamento crítico e fortalecer o protagonismo dos profissionais envolvidos.

**Conclusões:**

O fluxograma, de caráter multidirecional e participativo, promoveu maior sensibilização dos profissionais, aprimorou a comunicação intersetorial e fortaleceu a articulação entre os serviços de Saúde do Trabalhador e Saúde Mental, facilitando a identificação e encaminhamento de casos suspeitos.

## INTRODUCTION

Work-related mental disorders (WRMDs) are mental health conditions triggered by factors present in the work environment,^1^ characterized by symptoms such as irritability, insomnia, fatigue, and difficulty concentrating. These manifestations may be transient or persist for long periods, leading to reduced productivity across age groups and causing distress for the individual, their family, the workplace, and the wider community.^2^

In recent decades, work processes have undergone profound changes, influenced by globalization and new forms of work organization.^2^ In Brazil’s Unified Health System (SUS), WRMDs are conditions subject to compulsory notification in the Notifiable Diseases Information System (SINAN). Between 2007 and 2017, 8,474 cases of WRMDs were reported in SINAN/SUS, of which 65% were referred to a Psychosocial Care Center (CAPS) or another specialized service.^3,4^

Workers with suspected WRMDs must have the causal link established in a timely manner and, once the diagnosis is confirmed, should receive psychological support and appropriate care to prevent severe sequelae and reduce suffering.^4^ WRMDs are often disabling conditions and are associated with absenteeism and decreased productivity.^2^

During activities of an outreach project linked to the Education through Work for Health Program (PET-Saúde) at the Universidade Federal do Vale do São Francisco (Univasf), conducted in the city of Juazeiro, Bahia, Brazil, outreach (extension à outreach) students identified that CAPS, Basic Health Units, and the Reference Center for Occupational Health (CEREST) did not have a flowchart to guide the management of suspected or confirmed WRMD cases. In response to this gap, the project sought to raise awareness about the issue and to develop a flowchart that would represent, in a simple and ordered manner, the steps of the care pathway, supporting more effective decision-making and better work organization.^4^

This study emerged from the experience of the PET-Saúde group, a Ministry of Health initiative, with the support and participation of professionals from CAPS and CEREST of the Municipal Health Department of Juazeiro. The objective is to report the experience of students and faculty in the participatory construction of a flowchart for the management of suspected WRMD cases, involving teams from specialized Occupational Health and Mental Health services in the city.

## METHODS

This experience report aimed to describe the participatory construction of a flowchart for the management of suspected cases of WRMD, involving students, faculty, and professionals from specialized Occupational Health and Mental Health services of the Municipal Health Department of Juazeiro. The experience took place between January and July 2023, within the extension project “Strengthening mental health surveillance and psychosocial care for health workers in the city of Juazeiro - Bahia,” conducted by two health-trained faculty members and eight students from the Medicine, Nursing, and Psychology programs at Univasf, all participants in the PET-Saúde and linked to the Municipal Health Department of Juazeiro.

To achieve the proposed objective, professionals working in CAPS (CAPS I, CAPS II, and CAPS Alcohol and Drugs [CAPS AD]) and in the CEREST were included, as these sectors are reference services for mental health and occupational health actions, respectively.

CAPS I provides care for individuals with mental disorders such as depression, anxiety, and psychoses; CAPS II offers care for people with severe and persistent mental disorders; and CAPS AD is dedicated to the follow-up of users of alcohol and other drugs. These services have multidisciplinary teams composed of psychiatrists, nurses, psychologists, and social workers. CEREST provides specialized care for workers affected by work-related diseases and conditions, with a team that includes an occupational physician, an occupational health nurse, a psychologist, an occupational safety technician, and a physical therapist.

The construction of the flowchart was planned and implemented in distinct phases, with the additional aim of fostering collective awareness regarding mental health surveillance and psychosocial care for workers. The process comprised five stages conducted through active-learning methodologies in workshops using Problem-Based Learning. The problematization was guided by the Charles Maguerez Arch Method, which includes five phases: observation of reality, identification of key points, theorization, formulation of solution hypotheses, and application to reality.^5^

These stages were adapted to the extension-project context as a health-education strategy, encouraging participation and collective dialogue. Activities were carried out with the participation of the entire PET-Saúde group and included: i) technical visits to specialized services, CAPS and CEREST, to bring teams closer and to learn about work routines; ii) presentation and discussion of the Mental Health and Work Care Protocol and the SINAN form^6^ in a discussion circle, focusing on the identification of gaps; iii) collective analysis of the existing care pathway, identifying weaknesses in communication between services; and iv) shared construction of the care flowchart, using active methodologies to stimulate critical thinking and strengthen the leadership role of professionals.

With regard to ethical considerations, because this is an experience report, submission to a research ethics committee was not required, as the study describes the work experience of CAPS and CEREST professionals in Juazeiro and did not involve the collection of images or identifiable participant data.

## RESULTS

During the visits to the CAPES, several challenges were identified, particularly because psychiatrists did not participate in the initial meetings. As a result, two additional meetings with each physician were required, together with the PET-Saúde team, to present the Mental Health and Work Care Protocol and the SINAN notification form for cases of WRMD.

At these meetings, it became evident that the protocol was unknown to all CAPS professionals, in contrast to the CEREST team. Another concerning finding was that few CAPS professionals were familiar with the specific WRMD notification form, whereas at CEREST this knowledge was shared among all staff. Professionals were then asked to read these materials and describe how they would manage suspected cases of WRMD in their respective services.

The discussion circle highlighted two key points for understanding the difficulties in managing suspected WRMD cases: the lack of a structured intersectoral care network and the absence of a specific flowchart for this condition. The activity fostered deeper reflection, including on possible mistakes made as a result of limited knowledge of the protocol and the SINAN form.

To broaden learning and qualify the debate, the extension students proposed the analysis of everyday situations based on the services’ real context. This strategy encouraged professionals to recognize weaknesses and challenges in the process of identifying workers with suspected WRMD. This stage promoted knowledge exchange between services, strengthened intersectoral coordination, and raised awareness among teams about the importance of proper case recording and referral.

Building on the reflections developed in the discussion circles on the Mental Health and Work Care Protocol, the SINAN form, and the relevance of intersectoral action, the team began constructing a proposed care flowchart for cases of WRMD. The aim was to create a tool that would also include Primary Health Care (PHC) professionals, given their role as the entry point to the health-care network and, consequently, to workers’ health services.

However, because the PET-Saúde project ended, it was not possible to conduct visits to Basic Health Units and Family Health Units, and this stage was assigned to CEREST.

**Figure 1:**
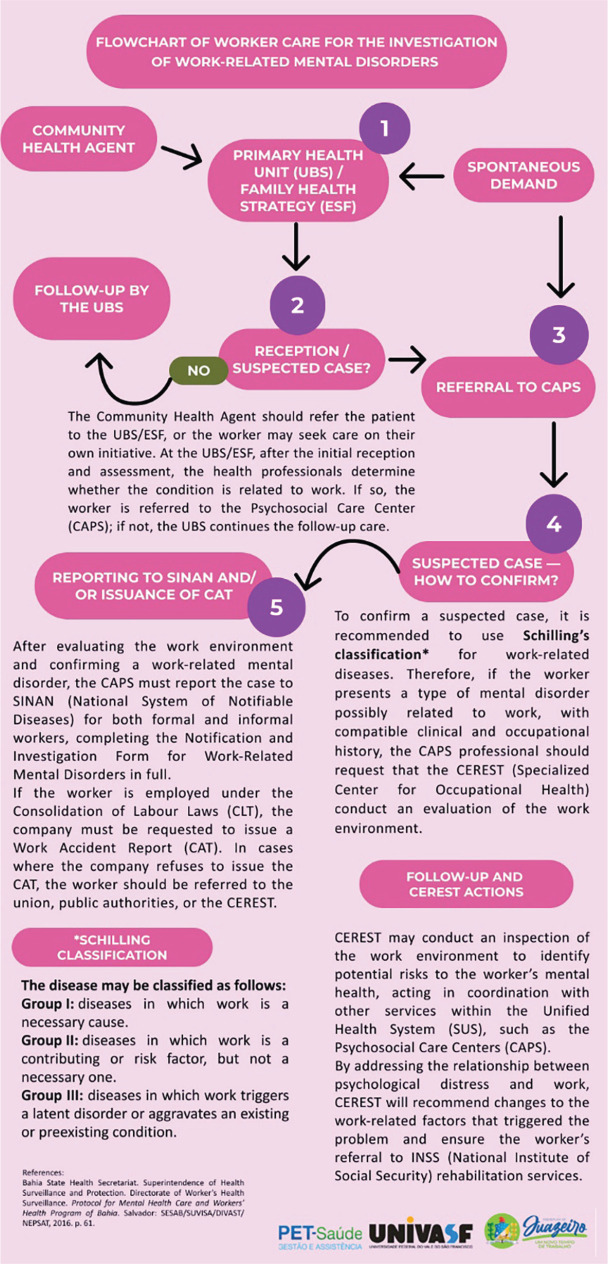
Care flowchart for the investigation of work-related mental disorders in the city of Juazeiro, state of Bahia, Brazil, 2025. Source: Prepared by the authors.

The meetings dedicated to the construction of the flowchart provided the basis for outlining a care model that is easy to understand for both professionals and users of occupational health and mental health services. The specific care flowchart for the investigation of cases of WRMD is presented in Figure 1.

## DISCUSSION

The relationship between work and mental health is highly complex, as it depends on how workers perceive aspects of their work environment and labor processes, which may be experienced positively or negatively. Work can generate satisfaction and benefits or, conversely, distress and illness.^7^ Understanding these effects requires considering personal life context, environmental conditions, and work organization. Frequently, the failure to establish the causal link between psychological distress and work activity results from insufficient integration among health networks and services.^8^

In this context, the lack of familiarity among CAPS professionals with the Mental Health and Work Care Protocol and with the WRMD notification form highlights a significant gap in managing these conditions. These instruments are essential for early identification, reporting, and coordination between mental health and occupational health services, as recommended by the Ministry of Health.^6^ Insufficient knowledge of these tools compromises clinical management, surveillance, reporting, and implementation of preventive actions in work environments.^6^

The findings of this study point to two essential issues in the care of workers with WRMDs. First, the absence of specific care pathways may lead to failures in case management, mainly due to unfamiliarity with the Mental Health and Work Care Protocol and the SINAN notification form. This finding reinforces the need for integration between services and continuous training of health professionals to ensure the quality of surveillance and care offered to workers.^1-9^

Furthermore, the analysis of real-life service scenarios, combined with knowledge exchange among professionals, proved to be an effective strategy for strengthening the team’s critical thinking regarding the challenges of care pathways. This practice aligns with active-learning methodologies and the promotion of collective reflection on mental health care in the work context.^10,11^

The exchange of knowledge enabled meaningful articulation between teaching and service, bringing together the University and Psychosocial Care Network devices, such as CAPS and CEREST. This integration allowed professionals to reflect on their roles and take an active part in reorganizing the care pathway. Studies on active methodologies indicate that dynamics fostering reflection based on concrete practice encourage changes in work processes.^12^

The collective construction of the flowchart thus represented a moment of critical reflection and articulation across different levels of care and areas of expertise. The use of discussion circles as structured spaces for dialogue encouraged reflection on service practices and processes, promoted intersectoral collaboration, and strengthened the understanding of the work-related mental health-disease process. This strategy fosters active listening, experience sharing, and collaborative work among professionals from different fields.^13^

In this context, the importance of applying the Schilling Classification was highlighted, as it guides the identification of the causal link between work and illness, distinguishing whether work is a necessary cause, a contributing factor, or an aggravating element.^14,15^ Using this instrument, together with integration between CAPS and CEREST, helps teams recognize and manage suspected cases of WRMD more effectively.

This approach strengthens collaborative practices and promotes closer coordination between services, both essential for comprehensive care.^15^ It also enables professionals to understand the points of articulation between Mental Health and Occupational Health, visualizing the ideal pathway for managing suspected WRMD cases. This strategy integrates learning into work processes and supports the transformation of practices based on the real needs of the population.^16^

Ensuring comprehensive care for workers with WRMDs requires coordinated action across surveillance, clinical care, and technical support within the health-care network. This coordination is primarily the responsibility of CEREST, which plays a central role in occupational health surveillance, specialized care, and technical support for the organization of actions within the territory. CEREST is the reference point for investigating work-related health conditions.^15^

CAPS, in turn, holds a fundamental position within the Psychosocial Care Network, providing continuous mental health care, clinical management, rehabilitation, and psychosocial support. This service must work in an integrated manner with PHC and CEREST to ensure proper establishment of the causal link and adequate follow-up of cases.^17^ National guidelines and care pathways emphasize the need for integration among CEREST, CAPS, and PHC through clear care flows and the use of tools such as protocols and notification forms, which are essential for identifying, reporting, and preventing WRMDs as well as avoiding care discontinuity.^16^

Failure to ensure such integration may result in fragmentation of care and lack of continuity in case follow-up. Effective communication among CEREST, CAPS, and PHC is therefore indispensable to guarantee comprehensive monitoring of workers, from identification of the condition to treatment and rehabilitation, thus ensuring integrality and care resolution.

In this context, it is important to recognize that work environments and processes may act as determining or aggravating factors in the development of mental disorders. Evidence indicates that work conditions such as overload, intense pace, lack of social support, and job insecurity influence the occurrence of WRMDs, compromising workers’ mental health and affecting their social and family relationships.^18,19^

Additionally, many workers rely on the public health system, reinforcing the need for PHC to incorporate mental health and act in coordination with the broader support network. Challenges, however, remain - particularly insufficient training and weak communication among points of care.^20^ Using support tools such as flowcharts may strengthen work processes in PHC, offering greater safety and problem-solving capacity to professionals, expanding their understanding of the work-related mental health-disease process, and enhancing collaborative care practices.^9^ Still, improving care for workers requires reorganizing the entire network, not only PHC.

## CONCLUSIONS

The participatory construction of a flowchart for the management of suspected WRMD cases highlighted the importance of integration among CEREST, CAPS, and PHC. This initiative fostered knowledge exchange, broadened understanding of the care pathway, and contributed to more timely identification and referral of cases.

This study demonstrates that tools such as flowcharts strengthen PHC’s problem-solving capacity, improve worker care, and promote intersectoral integration. These findings underscore the need for continuous professional training and policies that ensure comprehensive and coordinated occupational mental health care.
